# The Maternal Diet, Gut Bacteria, and Bacterial Metabolites during Pregnancy Influence Offspring Asthma

**DOI:** 10.3389/fimmu.2017.00365

**Published:** 2017-03-31

**Authors:** Lawrence E. K. Gray, Martin O’Hely, Sarath Ranganathan, Peter David Sly, Peter Vuillermin

**Affiliations:** ^1^Barwon Infant Study, School of Medicine, Deakin University, Geelong, VIC, Australia; ^2^Child Health Research Unit, Barwon Health, Geelong, VIC, Australia; ^3^Respiratory Diseases, Infection and Immunity Theme, Murdoch Children’s Research Institute, Parkville, VIC, Australia; ^4^Department of Respiratory and Sleep Medicine, Royal Children’s Hospital, Parkville, VIC, Australia; ^5^Department of Paediatrics, University of Melbourne, Parkville, VIC, Australia; ^6^Child Health Research Centre, The University of Queensland, Brisbane, QLD, Australia

**Keywords:** microbiome, respiratory, short-chain fatty acids, diet, maternal, offspring, asthma

## Abstract

This review focuses on the current evidence that maternal dietary and gut bacterial exposures during pregnancy influence the developing fetal immune system and subsequent offspring asthma. Part 1 addresses exposure to a farm environment, antibiotics, and prebiotic and probiotic supplementation that together indicate the importance of bacterial experience in immune programming and offspring asthma. Part 2 outlines proposed mechanisms to explain these associations including bacterial exposure of the fetoplacental unit; immunoglobulin-related transplacental transport of gut bacterial components; cytokine signaling producing fetomaternal immune alignment; and immune programming *via* metabolites produced by gut bacteria. Part 3 focuses on the interplay between diet, gut bacteria, and bacterial metabolites. Maternal diet influences fecal bacterial composition, with dietary microbiota-accessible carbohydrates (MACs) selecting short-chain fatty acid (SCFA)-producing bacteria. Current evidence from mouse models indicates an association between increased maternal dietary MACs, SCFA exposure during pregnancy, and reduced offspring asthma that is, at least in part, mediated by the induction of regulatory T lymphocytes in the fetal lung. Part 4 discusses considerations for future studies investigating maternal diet-by-microbiome determinants of offspring asthma including the challenge of measuring dietary MAC intake; limitations of the existing measures of the gut microbiome composition and metabolic activity; measures of SCFA exposure; and the complexities of childhood respiratory health assessment.

## Introduction

The human microbiome and its host form a complex symbiosis, and the gut microbiome is the primary interface for this relationship, harboring the most diverse array of microorganisms found in the human body (1). This diversity has been dramatically altered by diet and the modern environment (2). Changes in the human microbiome may be contributing to the rise of non-communicable diseases in developed societies, including childhood respiratory diseases such as asthma. These conditions pose a major health burden and have lifelong effects, with childhood asthma and poor early lung growth associated with respiratory disease in adulthood (3).

There is growing evidence that the maternal diet and gut bacteria influence offspring immune function and respiratory health (4). A number of mechanisms have been identified to explain the transplacental effects of maternal bacteria on the developing fetal immune system. These include bacterial exposure of the fetoplacental unit; immunoglobulin-related transplacental transport of gut bacterial components; cytokine signaling producing fetomaternal immune alignment; and immune programming *via* metabolites produced by gut bacteria. The role of gut bacteria and their metabolites in the development of fetal immune tolerance and subsequent offspring asthma is of particular interest.

Maternal gut bacteria are influenced by maternal diet, particularly intake of certain fibers and carbohydrates that have been collectively termed microbiota-accessible carbohydrates (MACs). Short-chain fatty acids (SCFAs) are the major metabolite produced by the gut bacteria from MACs. There is compelling evidence from mouse models linking variations in maternal dietary intake of MACs, gut bacteria, and SCFA production in the development of offspring asthma. These findings are yet to be confirmed in human studies. The aims of this review are to assess the current evidence regarding the influence of maternal dietary MACs and gut bacterial exposures during pregnancy on the developing fetal immune system and subsequent offspring asthma and discuss the considerations for future studies in this emerging field.

## Part 1

### Evidence that Maternal Bacterial Exposures during Pregnancy Are Associated with Offspring Asthma

Maternal exposure to a farming environment during pregnancy is associated with diverse bacterial experience and reduced risk of asthma in offspring (5). In the Protection against Allergy-STUdy in Rural Environments (PASTURE) study, von Mutius et al. measured levels of bacterial endotoxin in the home of farming and non-farming families, demonstrating that higher exposure was associated with reduced asthma incidence (6). The Prevention of Allergy Risk factors for Sensitization In children related to Farming and Anthroposophic Lifestyle (PARSIFAL) (7) and the Multidisciplinary Study to Identify the Genetic and Environmental Causes of Asthma in the European Community Advanced Study (GABRIELA) (8) are two important European cohorts comparing children living on farms with those living in suburban areas. Both the PARSIFAL and GABRIELA cohorts showed an association between a farming environment and increased bacterial prevalence in mattress dust (9, 10). Ege et al. reported results from both cohorts, identifying that children living on farms had a lower prevalence of asthma and other atopic disease in comparison to those from suburban areas (11). Further analysis of these studies has indicated that the effect on asthma is largely explained by exposure to cows, straw, and consumption of raw cow’s milk (12); although unspecified, livestock exposure is also associated with a lower prevalence of asthma (13). The effect of a farming environment and livestock exposure on asthma has been supported by recent evidence comparing children from the Amish and Hutterite groups in the United States, both isolated agrarian communities of genetically similar European descent. Differences between the groups were correlated with farming practices, with high dairy farm and livestock exposure in the Amish associated with lower incidence of asthma and allergic sensitization (14).

There is evidence that the “farm effect” (12) is not confined to childhood but acts on the developing fetal immune system during pregnancy. A farming environment has been associated with increased number and efficiency of regulatory T lymphocytes (Tregs) (15) as well as an altered cytokine profile in cord blood (16). Finally, the PASTURE study has demonstrated that maternal exposure to a farming environment is associated with reduced offspring asthma, although this effect is enhanced by early-life farm exposure (17). The “farm effect” provides strong evidence that maternal bacterial exposure during pregnancy influences the fetal immune system and subsequent risk of offspring asthma.

Prebiotic and probiotic supplementation during pregnancy may alter maternal gut bacteria and influence maternal immune function and offspring asthma. These supplements represent a growing worldwide industry worth billions of dollars (18), despite insufficient evidence of their purported benefits in many cases (19). A prebiotic is a “selectively fermented ingredient that allows specific changes, both in the composition and/or activity in the gastrointestinal microbiota thus conferring benefit …” (20). The short-chain galactooligosaccharides (scGOS) and long-chain fructooligosaccharides (lcFOS) are human milk oligosaccharides that have attracted research interest for their potential use in infant formula. Prebiotic human milk oligosaccharides have immune-modulating effects. Acidic milk oligosaccharides can alter the production of many cytokines (21), and scGOS/lcFOS have been shown in other allergic and autoimmune conditions to act *via* galectin-9 to suppress immune responses, promoting interferon-gamma production by T-helper 1 (Th1) cells and inducing Tregs (22–24). The galectins and other glycan-binding receptors in the intestinal epithelium such as C-type lectins and siglecs are important potential immune modulators. These receptors bind many glycans including the human milk oligosaccharides and other carbohydrates and are implicated in the putative benefits of prebiotic supplementation (25). scGOS/lcFOS supplementation during pregnancy may also alter the fecal microbiome, promoting an increase in maternal bifidobacteria (26). There is preliminary evidence that increased maternal exposure to scGOS/lcFOS in pregnancy may be associated with reduced offspring wheeze. Hogenkamp et al. supplemented the feed of Balb/c mice with scGOS/lcFOS during pregnancy, then fed offspring with a normal diet, and observed lower lung resistance in response to methacholine challenge (27). In humans, scGOS/lcFOS have been shown to reduce recurrent wheeze in high-risk children supplemented from birth (28); however, similar effects on offspring of mothers supplemented during pregnancy have not been reported, and the comprehensive 2016 World Allergy Organization review found a lack of evidence to support the use of prebiotics during pregnancy (20).

Probiotics are products containing live microbes designed to confer a health benefit. Studies of *Lactobacillus rhamnosus* GG supplementation demonstrated maternal and infant fecal colonization with the strain (29, 30), although the small number of subjects in each trial limits the generalizability of these findings. A recent meta-analysis of studies in healthy adults found a lack of evidence for an effect of probiotic supplementation on fecal microbiota (31). There is evidence suggesting that probiotic supplementation influences the maternal immune system. A study of *Lactobacillus casei* DN11401 supplementation during pregnancy found an association between supplementation and changes in maternal serum natural killer cells and decreased breast milk tumor necrosis factor alpha (32). Other studies have demonstrated changes in breast milk cytokines following probiotic supplementation (33). Despite evidence of alterations in fecal bacteria and effects on immune development, several clinical trials (33–44) and a well-conducted meta-analysis (45) have not found an association between probiotic supplementation in pregnancy and childhood asthma. The major limitation of trials of probiotic supplementation during pregnancy is that supplementation is not commenced until late in pregnancy, after a critical window of fetal immune development (46). The results of a planned trial of supplementation with *L. rhamnosus* HN001 from the first trimester of pregnancy (47) may provide clearer evidence for associations between probiotic supplementation in pregnancy, fetal immune development, and offspring asthma.

Antibiotic use is associated with alterations in fecal bacteria and increased risk of asthma. Antibiotics rapidly alter fecal bacteria in adults and children, thereby leading to reduced richness and diversity (48, 49). A Finnish cohort study of 236 children found macrolide antibiotic use was associated with alterations in specific bacterial phyla, with a reduction in phylum Actinobacteria and an increase in phylum Bacteroidetes (50). Reductions in bacterial richness were found to persist for more than 2 years after exposure. This study also demonstrated that early macrolide use was associated with subsequent increased risk of asthma. An increased risk of asthma following childhood antibiotic use has also been reported for cephalosporins (51) and for antibiotics in the International Study of Asthma and Allergies in Childhood Phase III study (52). Maternal antibiotic use during pregnancy may influence offspring asthma. A study utilizing the West Midlands General Practice Research Database, a large birth cohort of 24,690 children, demonstrated an association between maternal antibiotic use during pregnancy and increased risk of allergic disease, with two or more courses of antibiotics during pregnancy carrying a hazard ratio of 1.68 for offspring asthma (53). This finding has since been supported in large studies using the Copenhagen Prospective Study on Asthma in Childhood (COPSAC) cohort and the Danish Birth Registry, which demonstrated that maternal antibiotic use during pregnancy was associated with a dosage-related increase in risk of offspring asthma (54–58). There is clear evidence that maternal antibiotic use in pregnancy leads to altered fecal bacterial phyla, reduced fecal bacterial species diversity, and increased risk of offspring asthma.

Summary of evidence in this section is found in Table 1.

**Table 1:** **Evidence that maternal bacterial exposures during pregnancy are associated with immune programming and offspring asthma**.

Bacterial exposure	Result category	Result	Reference
Farm effect	Bacterial exposure	Increased endotoxin exposure in house	Protection against Allergy-STUdy in Rural Environments study (6)
		Increased bacterial prevalence in mattress dust	Prevention of Allergy Risk factors for Sensitization In children related to Farming and Anthroposophic Lifestyle (7, 9); Genetic and Environmental Causes of Asthma in the European Community Advanced Study (8, 10)
	Fetal immune programming	Alterations in cord blood cytokines	(16)
		Increased regulatory T lymphocytes (Tregs)	(15)
	Asthma	Decreased childhood asthma	(5) (review), (11, 14)
		Decreased offspring asthma	(17)
Prebiotic short-chain galactooligosaccharides/long-chain fructooligosaccharides	Fecal microbiome	Maternal supplementation associated with increased bifidobacteria	(26)
	Immune function in other autoimmune conditions	Increased T-helper 1 interferon-gamma production Treg induction	(23, 24)
			(22, 24)
	Asthma	Maternal supplementation associated with improved offspring lung function in mice	(27)
		Supplementation in infants from birth associated with decreased childhood wheeze	(28)

Probiotics	Fecal colonization	Maternal transfer of colonization to offspring	(29, 30)
		No maternal transfer of colonization	(31) (meta-analysis)
	Immune function	Alterations in breast milk cytokines	(32, 33)
	Asthma	No associations found maternal supplementation with offspring asthma	(45) (meta-analysis)

Antibiotics	Fecal microbiome	Reduced richness and diversity	(48–50)
	Asthma	Childhood use associated with increased childhood asthma	(50–52)
		Maternal use during pregnancy associated with increased offspring asthma	(53, 56)

## Part 2

### Putative Mechanisms for Maternal Gut Bacterial Influence on Immune Programming and Offspring Asthma (Figure 1)

**Figure 1:**
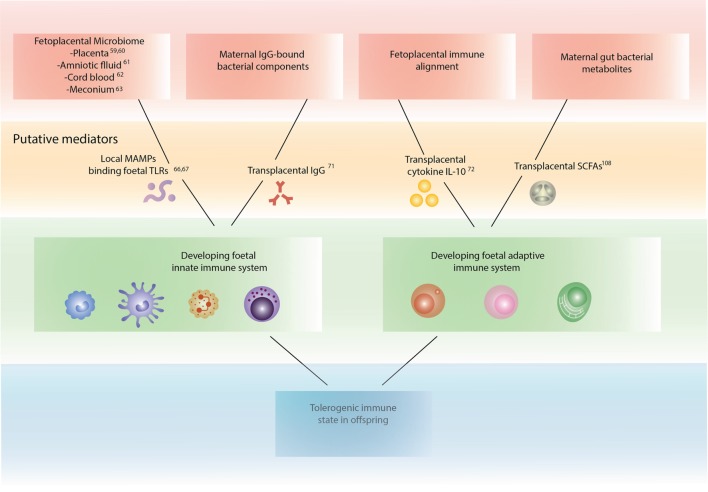
**Mechanisms by which maternal bacteria may influence fetal immune development**.

#### Fetoplacental Microbiome

Bacteria have been identified throughout the fetoplacental unit that may interact with the developing fetal immune system. Chorioamnionitis, an infection of the placenta, membranes, or amniotic fluid associated with prolonged rupture of the membranes, is a cause of preterm birth and neonatal infection. Until recently, this condition was thought to indicate bacterial invasion of previously sterile tissues. Evidence of bacterial colonization has now been found in the placenta (59), placental membranes (60), amniotic fluid (61), and cord blood (62). Bacterial genetic material has been identified in the placental membranes of preterm and term deliveries with and without prior labor, indicating bacterial colonization of these tissues prior to delivery (60). Bacteria may colonize the fetoplacental unit early in pregnancy, as bacterial genetic material has been identified in the amniotic fluid following amniocentesis (61), although bacteria have not been cultured from this site in humans. Evidence from mice indicates that meconium and cord blood may also be colonized with bacteria during pregnancy. Humanized mouse mothers were orally inoculated with a labeled strain of *Enterococcus faecium* from human breast milk, and the organism subsequently cultured from amniotic fluid (62) and offspring meconium (63). Bacterial genetic material has also been recently identified in the human placenta (59), although this finding was questioned on the basis of low read counts and possible contamination with environmental bacteria at the time of collection (64). Bacteria have not been successfully cultured from the placenta to date (65). The presence of bacterial genetic material at these sites remains an important finding, with evidence that bacterial DNA may be sufficient to effect fetal immune modulation. Unmethylated CpG motifs in bacterial DNA have been demonstrated to exert immunostimulatory effects *via* toll-like receptor (TLR) 9 (66, 67). TLRs are expressed in many leukocytes and have an established role in innate immune stimulation (68). TLRs are also important in adaptive immunity, with dendritic cells releasing regulatory cytokines in response to TLR binding to promote a Th1 phenotype (68) and a number of TLRs identified on Tregs including TLRs 4, 5, 7, and 8 (69). Early and persistent exposure of the fetoplacental unit to maternal bacteria and bacterial genetic material during pregnancy is likely to be an important mechanism of fetal immune programming. This effect may be enhanced by mechanisms described below that increase transplacental transport of bacterial components, cytokines, and bacterial metabolites.

#### Maternal Immunoglobulin G (IgG)-Bound Bacterial Components

Transplacental transport of gut bacterial components bound to maternal immunoglobulin influences the developing fetal immune system and reduces inflammatory responses in offspring. Studies in mouse models have demonstrated that transplacental transport of allergen-specific IgG is associated with reduced offspring asthma (70). There is now evidence to support transplacental transport of bacterial components bound to maternal IgG. Gomez de Aguero et al. transiently colonized the gut of a pregnant germ-free mouse with a modified strain of *E. coli* HA107 that could not persist, returning the mice to a germ-free state prior to delivery (71). They observed no changes in offspring adaptive immune cell numbers but found increases in intestinal innate lymphoid and mononuclear cells, suggesting that even transient colonization was linked to fetal immune modulatory effects. Serum from transiently infected mothers was transferred to unexposed pregnant mothers, with a subsequent increase in offspring intestinal innate lymphoid cells. When the serum was depleted of IgG prior to transfer, this effect was lost, indicating the importance of IgG-mediated transfer of bacterial components. An increase in intestinal mononuclear cells was observed in offspring regardless of serum IgG depletion, suggesting that antibody-mediated transfer is part of a group of transplacental signals responsible for fetal immune programming.

#### Fetoplacental Immune Alignment

Transplacental cytokine signaling promotes maternal–fetal immune alignment. Santner-Nanan et al. examined serum from parents and their offspring, comparing the percentage of peripheral Tregs at term in mother–child dyads with that in father–child dyads (72). A high correlation was found between mother–child dyads but not father–child dyads. A high correlation was also identified for interleukin (IL)-10 levels, as well as an upregulation of IL-10 receptor alpha in the mother–child dyads, suggesting that the mechanism for Treg alignment is transplacental signaling by IL-10. Cytokine signaling appears to be another important mechanism influencing the developing fetal immune system.

#### Maternal Gut Bacterial Metabolites

Transplacental action of gut bacterial metabolites influences many host functions, including fetal immune development. Gut bacteria produces numerous metabolites that are critical mediators of host function, influencing processes such as immune modulation, inflammation, epigenetic changes, and energy production (73, 74). SCFAs derived from MACs are a key group of metabolites involved in immune modulation and have attracted much research attention. The three most common SCFAs produced in the gut are acetate, propionate, and butyrate (75), of which the majority are butyrate. SCFAs are produced in abundance as a by-product of bacterial carbohydrate fermentation and are a key energy source for the human host, supplying approximately 10% of total energy requirement (76). Further discussion of SCFAs in fetal immune development and subsequent offspring asthma is detailed in Part 3 of this review (77).

Summary of evidence in this section is found in Table 2.

**Table 2:** **Putative mechanisms for maternal gut bacterial influence on immune programming and offspring asthma**.

Putative mechanism	Description	Reference
Fetoplacental microbiome	Bacteria present throughout the fetoplacental unit	(59), (64) (placenta), (60) (placental membranes), (61) (amniotic fluid), (62) (cord blood)
	Immunostimulatory effects of bacterial DNA	(66, 67)

Maternal immunoglobulin G-bound bacterial components	Transplacental transport of bacterial components from modified *E. coli* in mice	(71)
Fetoplacental immune alignment	Correlation between regulatory T lymphocytes (Tregs) and interleukin-10 in mother–child dyads	(72)

Short-chain fatty acids	Tolerogenic immune state	(90–95)
	Increased Treg population and function	(103–106)
	Offspring asthma	(108)

## Part 3

### Evidence that Maternal Diet and Bacterial Metabolites during Pregnancy Influence Offspring Asthma

#### Diet Influences Fecal Bacterial Composition

Short-term dietary change alters fecal bacterial composition rapidly, but reversibly, while long-term dietary change is associated with irreversible alterations. Subjects fed either a plant or animal-based diet exclusively for 5 days had alterations in fecal bacterial composition, with an animal-based diet leading to a relative reduction in bacteria from phylum Firmicutes (78). This change was rapid, occurring within the first day of a new diet; however, fecal bacteria reverted its original composition 2 days after ceasing. This finding is supported by other human studies indicating short-term dietary changes have temporary effects on fecal bacterial composition (79). However, elegant work by Sonnenburg et al. has suggested that long-term dietary change may alter fecal bacterial composition irreversibly (80). Mice were fed restricted diets across several generations, demonstrating increased loss of bacterial diversity with each successive generation. Bacterial diversity loss was only partly reversible with a return to a broader diet. These findings are consistent with changes in fecal bacterial composition identified in modern industrialized societies. Studies in humans comparing the fecal bacteria of hunter–gatherer with modern societies have consistently found increased microbial diversity in the hunter–gatherer groups, considered to reflect differences in dietary staples (2, 81, 82). Nakayama et al. have recently demonstrated that a widespread geographic distribution of populations is not required for these differences to emerge (83). Fecal bacterial composition in children from different Asian countries was analyzed, and associations were identified between fecal bacterial composition and either regional or national dietary differences. Differences in fecal bacterial composition were also found between rural and metropolitan populations within Thailand, with the authors proposing that differences in dietary fruit intake may explain these. Evidence from mouse and human studies has demonstrated that diet influences fecal bacterial composition. Dietary changes that affect fecal bacterial composition may also affect the production of bacterial metabolites such as SCFAs.

Short-chain fatty acids are produced by the fermentation of MACs. MACs may be consumed in the host diet, produced by the host or by other bacteria. The bulk of these carbohydrates are found in dietary fiber that resists digestion by the host, although MACs are found in small amounts in many foods (84). A proportion of MAC is also derived from the mucous layer of the gut, a key bacterial substrate in periods of low dietary MAC availability (85, 86). Until recently, studies have used the terms dietary fiber or fermentable fiber as a catchall to describe this “microbiota food” (86); however, this terminology is problematic. First, it assumes the presence of the appropriate microbial species to perform the fermentation, which may not exist in a limited bacterial population. Second, it implies that fiber intake is an adequate surrogate marker of MAC intake; however, total dietary MAC intake is not currently accurately quantified by dietary surveys such as the Food Frequency Questionnaire (FFQ) (86). Finally, there are many other substrates that contribute these carbohydrates that are not considered fibers, such as resistant starches or human milk oligosaccharides (84). To address these difficulties, Sonnenburg et al. have proposed the term MAC (80, 87). This is preferred as it does not have an implied link to total dietary fiber and highlights the importance of these substrates in determining the gut bacterial species mix (87).

Increased dietary MACs promote a gut microbiome characterized by SCFA-producing bacteria and increased fecal SCFAs. In their landmark study, De Filippo et al. compared the fecal bacterial composition of children living in a village in Burkina Faso with the fecal bacterial composition of a group of Italian children (88). The Burkina Faso village was chosen for its similarity to a pre-agrarian, hunter–gatherer society, whose high dietary fiber intake closely resembled that of the Neolithic period prior to widespread cultivation of food. Feces from children in the African group had markedly increased phylum Bacteroidetes and reduced phylum Firmicutes in comparison to European counterparts. De Fillipo et al. also indicated that the high-fiber diet of the Burkina Faso group was associated with increased fecal SCFAs, with a fourfold increase in fecal butyrate and propionate. Analysis of fecal bacterial composition of other hunter–gatherer populations in Malawi and Venezuela has found a high number of bacterial genes associated with digestion of MACs (81). Following on from these observational studies, *in vitro* experiments with a model human colon populated with fecal bacteria have demonstrated an association between increased proportions of genus Bacteroides and Prevotella (both from phylum Bacteroidetes) and propionate concentrations (89). Diet influences fecal bacterial composition, with a diet high in MACs associated with increased SCFA-producing phyla and increased fecal SCFAs.

#### SCFAs Produced by Maternal Gut Bacteria Influence Fetal Immune Development and Offspring Asthma

Short-chain fatty acids produced by maternal gut bacteria indirectly influence T lymphocytes to produce a tolerogenic immune environment in the offspring. SCFAs bind three G protein-coupled receptors [also termed free fatty acid receptors (FFARs)], GPR41/FFAR3, GPR43/FFAR2, and GPR109a (niacin receptor), of which only GPR43 is specific to leukocytes. SCFAs influence neutrophils and eosinophils by binding GPR43 (90) and through direct inhibition of histone deacetylase (HDAC), resulting in apoptosis (91). Butyrate and propionate have been shown separately to regulate dendritic cell function by this mechanism, reducing stimulation of T lymphocytes (92, 93), and the same pathway was found to operate in colonic inflammatory responses (94). Butyrate also stimulates the niacin receptor GPR109a with GPR109a-deficient mice exhibiting reduced IL-10-producing T lymphocytes (95).

Regulatory T lymphocytes produce a tolerogenic immune profile and promote a Th1 phenotype protective against the development of asthma (96). Traditionally, the adaptive immune system was described as either Th1 or Th2 dominant, with asthma associated with the Th2 phenotype. The Th2 phenotype is associated with increased IgE production, eosinophilia, and production of cytokines IL-4, IL-5, IL-9, and IL-13 (97). These cytokines activate an allergic inflammatory response to encountered aeroallergens and infections that produces the symptoms of asthma (98). Early studies identified a reduced population of serum Tregs in asthmatic patients (99, 100). Mouse models of allergic airways inflammation have shown that introduced Tregs inhibit disease development and suppress established disease (101). A key mechanism for Treg-mediated immune modulation and Th1 skewing is the transcription factor forkhead box p3 (Foxp3). In the presence of allergenic stimulation, Treg cells expressing high amounts of Foxp3 produce IL-10 and transforming growth factor beta, suppressing the activity of dendritic cells and other T-cells and subsequently reducing allergic responses (102).

Short-chain fatty acids directly influence Treg population and function. Acetate and propionate promote colonic Treg production with an associated increase in Foxp3 and IL-10 expression in these cells (103). This effect is mediated through inhibition of HDAC, resulting in increased acetylation of Foxp3 and associated increased stability and expression at cell surfaces. This mechanism has been identified in other studies in which mice fed butyrate and propionate increased the number of extrathymic (104) and colonic Tregs (105). SCFAs have both indirect and direct effects on T lymphocytes promoting a tolerogenic immune profile, with increased Treg populations and activity (106).

Mouse models have demonstrated an association between increased maternal dietary MACs, SCFA exposure during pregnancy, and reduced offspring asthma. Trompette et al. and Thorburn et al. demonstrated that increased maternal dietary MACs were associated with reduced severity of allergic airway inflammation in offspring (107, 108). Trompette et al. reproduced the mechanism of SCFA action through GPR43 and associated changes to dendritic cell population and T lymphocyte activity (107). The study by Thorburn et al. was unique in finding that pregnant mice exposed to a high MAC diet or acetate have offspring that appear protected from developing allergic airways disease and that this effect persists into adulthood (108). A follow-up small human component (*n* = 61) of the same study showed an association between reduced recalled maternal dietary fiber intake and reduced serum acetate levels. A separate component (*n* = 40) showed an association between serum acetate below median level and increased doctor visits for cough/wheeze and wheeze in offspring in the first year, although a reverse trend appears for the other SCFAs measured. The author’s choice of respiratory health outcome in the human components is notable. Two or more episodes of GP reported cough in the first year may be associated with maternal serum acetate but is unlikely to yield a distinct clinical phenotype that is predictive of subsequent risk of asthma (108). In demonstrating an association between maternal dietary MACs, antenatal exposure to SCFAs, and offspring asthma, these mouse experiments have suggested a possible target for interventions to reduce the burden of respiratory illnesses such as asthma; however, further human studies are required.

#### Other Maternal Dietary Factors during Pregnancy Are Associated with Immune-Modulating Effects and Offspring Asthma

Maternal exposure to increased vitamin D during pregnancy may influence fetal immune and lung development and reduces offspring wheeze. Vitamin D receptors are found in many immune cells (109), and early studies showed that vitamin D was able to promote specific Treg populations (110) as well as numerous other effects on innate immune pathways (111). The effect of maternal vitamin D on fetal immune development is less clear with recent studies finding that high maternal serum vitamin D associated with decreased Foxp3-expressing Tregs in cord blood (112). Any association found with offspring asthma is complicated by the effect of vitamin D on fetal lung development, with low vitamin D associated with reduced lung volumes in mouse models (113). Evidence from birth cohort studies indicates that high maternal dietary vitamin D intake is associated with reduced offspring wheeze (114–117). Recent well-designed, double-blind, randomized, control studies failed to find a similar association for vitamin D supplementation, although the control group in both cases was receiving the recommended daily dose of vitamin D throughout (118, 119). Maternal vitamin D exposure, either from diet or supplementation, influences fetal immune and lung development and the risk of offspring wheeze.

Maternal dietary vitamin E intake influences fetal immune and lung development and risk of offspring wheeze. Vitamin E has been found to have effects on many immune pathways, reducing inflammation (111). Vitamin E has tolerogenic effects on T lymphocytes, reducing their stimulation by interferon-gamma (120) and promoting their survival (121). Devereux et al. identified that high maternal dietary intake of vitamin E during pregnancy was associated with a decreased Th cell proliferative response in cord blood mononuclear cells (122). Vitamin E intake in pregnancy has been associated with reduced wheeze in children in a number of studies (123–126). Vitamin E also has effects on lung function, with an association between increased maternal vitamin E intake and higher offspring forced expiratory volume in 1 s (FEV_1_) at 5 years (124). Maternal exposure to decreased dietary vitamin E during pregnancy increases offspring cord blood mononuclear cell proliferation and is associated with reduced offspring wheeze and improved lung function.

Maternal intake of polyunsaturated fatty acids (PUFAs) may alter fetal immune development and is associated with offspring asthma. The most researched group of PUFAs is the N-3 (omega-3 fatty acids) which may promote a Th1 phenotype and stimulate Tregs and the N-6 (omega-6 fatty acids), associated with increased inflammation and Th2 skewing (127). It has been hypothesized that reductions in the ratio of dietary N-3:N-6 PUFAs in Western societies may be responsible for an increase in allergic disease (128). Evidence in mice suggests that maternal PUFAs may influence fetal immune function. van Vlies et al. fed mice mothers differing ratios of N-3:N-6 PUFAs and found that both a high and low ratio reduced offspring Th2 response (129). Studies measuring PUFAs in the serum of pregnant women found minimal evidence of an association between PUFA ratios and childhood respiratory outcomes (130, 131). Two similar studies by Nwaru et al. and Lumia et al. within the same study population examined dietary intake of PUFAs and found conflicting results. Nwaru et al. reported an association between low PUFA intake and allergic rhinitis but not asthma in offspring at 5 years (132). Lumia et al. reported an association between low PUFA intake and asthma at 5 years (133). Oily fish such as salmon and herring contain increased ratios of N-3:N-6; however, until recently, trials of supplementation with fish or fish oil during pregnancy had found only weak evidence of an association with reduced offspring asthma (134–139). In 2016, Bisgaard et al. reported from the COPSAC cohort that supplementation with N-3 PUFA during the third trimester of pregnancy was associated with a relative risk reduction for persistent wheeze or asthma in offspring of 30.7% (140), although there was no associations found with other allergic outcomes and the level of supplementation was 15 times greater than the expected average intake by pregnant mothers (141). Maternal supplementation with fish-derived N-3PUFAs may alter fetal immune development and is associated with reduced offspring asthma.

Summary of evidence in this section is found in Table 3.

**Table 3:** **Maternal dietary factors during pregnancy associated with immune modulation and offspring asthma**.

Dietary factor	Result category	Result	Reference
Vitamin D	Immune function	Vitamin D enhances regulatory T lymphocyte (Treg) activity	(110), (111) (review)
	Fetal immune programming	Increased maternal serum vitamin D associated with reduced offspring forkhead box p3-expressing Tregs	(112)
	Offspring asthma	Increased maternal dietary vitamin D intake associated with reduced offspring wheeze	(114–117)
		Maternal vitamin D supplementation not associated with offspring wheeze	(118, 119)

Vitamin E	Immune function	Vitamin E reduces inflammation	(111) (review)
		Vitamin E reduces T lymphocyte stimulation by interferon-gamma	(120)
		Vitamin E increases T lymphocyte survival	(121)
	Fetal immune programming	Increased maternal dietary vitamin E decreases T helper response in cord blood	(122)
	Offspring asthma	Increased maternal dietary vitamin E intake associated with reduced offspring wheeze	(123–126)
		Increased maternal dietary vitamin E intake associated with improved offspring lung function	(124)

Polyunsaturated fatty acids (PUFAs)	Fetal immune programming	Increased and decreased N-3:N-6 PUFA ratio associated with reduced offspring Th2 response in mice	(129)
	Offspring asthma	Maternal serum PUFAs not associated with offspring asthma	(130, 131)
		Increased maternal dietary PUFAs or supplementation with fish or supplementation with lower dosage of fish oil not associated with offspring asthma	(132–139)
		Maternal supplementation with high dosage of fish oil associated with reduced offspring asthma	(140)

## Part 4

### Challenges for Future Research into the Maternal Diet, Gut Bacteria, Microbial Metabolites, and Offspring Asthma

There are currently no validated methods to estimate dietary exposure to MACs. The FFQ is the most widely used measure of diet in the studies discussed in this review. FFQ allows an estimation of dietary exposures to many specific foods, food groups, or micronutrients; however, dietary exposure to MACs is more difficult to estimate. MACs defy traditional categorization into fibers or carbohydrates due to their ubiquity in small amounts across a range of foods and food types, and there is currently no validated method for this estimation (142). Establishing an accurate estimate of MAC exposure from diet remains the subject of current research efforts (143) and is critical to investigating any association between MACs and other outcomes.

Gut bacterial composition is inferred from the fecal microbiome. When assembling this review, we identified a single prospective study that examined the relationship between maternal fecal bacteria and offspring asthma as its primary outcome. Lange et al. examined fecal bacteria from 60 mothers during the third trimester and found evidence of an association between higher total aerobes and enterococci and infant wheeze (144). The small study size, use of a culture-dependent method, and assessment of only very early respiratory outcomes limits the interpretation of these findings. These limitations do not detract from the importance of demonstrating an association between maternal gut bacteria in pregnancy and offspring asthma using a culture-dependent method. Culture-independent methods based on bacterial genetic material have largely replaced such studies.

Current studies of gut bacteria use bacterial genetic material extracted from fecal samples and analyzed using next-generation techniques to determine a fecal microbiome. The microbiome is a detailed picture of the species, relative abundance, diversity, and metabolic functions of all the microbiota present in the gut, inferred entirely from genetic material collected from feces. There are two principal approaches to the analysis of the gut microbiome: shotgun metagenomic sequencing and 16S sequencing. Shotgun metagenomic sequencing extracts all the DNAs from a fecal sample, fragments it, and sequences on one of several available massively parallel sequencing (MPS) platforms. Resulting reads are then assembled into longer sequences and aligned to databases of known and predicted genes, thus inferring the functional potential of the microbiota and giving an approximation of the taxa present in the sample. At present, this approach suffers from a high cost per sample; but it is reasonable to think the price will decline in the next few years.

16S sequencing targets a few hundred bases of a gene believed to be common to all bacterial species, the 16S ribosomal subunit. Variability within this gene has been the gold standard for taxonomic classification for bacteria. The selected fragment of 16S rDNA is extracted from the DNA in a sample, amplified *via* PCR and usually ligated to barcode DNA oligomers for multiplex MPS sequencing. Resulting reads are demultiplexed, clustered into operational taxonomic units (collections of sequence that satisfy a similarity criterion designed to avoid spurious variation due to sequencing errors) and aligned to comprehensive databases of 16S rDNA sequences. 16S sequencing gives a putative taxonomic profile of the species present in the sample; however, functional information has to be inferred.

The fecal microbiome is highly likely to be representative of the gut bacterial composition in the lumen of the lower digestive tract but may not reflect the full complexity of human gut bacteria and may be contaminated with environmental bacterial genetic material or the genetic material of dead bacteria. Furthermore, as the fecal microbiome is only a proxy measure of the gut microbiota, absolute quantitative information is dubious. Relative quantitative comparisons between samples may be valid, provided samples have been stored and treated uniformly. Numbers of reads assigned to a taxon should be treated with caution as PCR biases, gene copy number variation, and variability in regions surrounding the formal “variable” regions can affect primer efficiency. Software tools comparing the composition of samples either use straightforward statistical tests for *a priori* hypotheses, more sophisticated statistical analyses (usually derived from techniques used in RNA-seq analysis, and perhaps sub-optimal for microbiome analysis) for discovery of differential abundances between experimental conditions, or biological outcomes, or use machine-learning approaches to detect more subtle patterns among bacterial composition, treatments, and outcomes. There is currently no consensus in the field as to which methods represent best practice, and there is substantial scope for more mature techniques.

Measurement of SCFA production by gut bacteria and SCFA exposure in humans is challenging. The majority of SCFAs produced are rapidly consumed by colonocytes. A unique study by Cummings et al. carried out in cadavers demonstrated that the concentration of SCFAs, particularly butyrate, significantly diminishes between the cecum and rectum (145). Colonocyte consumption of butyrate does not produce a specific marker or by-product to allow total butyrate consumption during gut transit to be measured. Colonocyte consumption is also dependent on cellular energy requirements that vary under different conditions. Subsequently, fecal SCFAs are a limited marker of total SCFA exposure. Serum SCFAs are also an inadequate measure of total SCFA exposure. Colonocyte consumption prevents the majority of butyrate reaching the serum and proportionally more acetate and propionate are found in the serum. Cummings et al. found significant differences in acetate and propionate concentrations at different blood sampling sites with the highest concentrations found in the portal vein and the lowest concentrations found in the peripheral veins indicating acetate and propionate are subject to first-pass hepatic metabolism (145). Acetate and propionate are primarily utilized by the liver in gluconeongenesis, along with many other substrates. SCFA utilization in gluconeogenesis does not produce a specific marker or by-product to allow total acetate and propionate consumption to be measured. Despite these limitations, measures of SCFAs in feces and serum are currently used to indicate total host SCFA exposure. Further research is needed into alternate methods to measure SCFA exposure, and the limitations of serum and fecal SCFA measurement must be considered when describing SCFA-related associations.

Defining offspring respiratory health phenotypes that predict asthma that persists into later life may be difficult in infancy and early childhood. Childhood respiratory health has been defined using a variety of outcome measures in the studies discussed, including parent-reported symptoms; medication use, hospital presentations or physician diagnosis; and lung function testing. These measures have not been utilized consistently and case definitions of wheeze and asthma vary throughout the studies. Only a small number of studies performed lung function testing, likely reflecting difficulty in obtaining accurate lung function measures in preschool children. Newer tidal volume techniques such as Multiple Breath Washout or Forced Oscillation Testing allow measurement in children from birth (146). Future studies must carefully consider the respiratory outcomes chosen and may benefit from tidal volume lung function techniques.

## Conclusion

There is evidence that antenatal interaction between maternal diet, gut bacteria, bacterial metabolites, and the developing fetal immune system influence offspring asthma. In mouse studies, these associations have been linked, providing evidence that dietary MACs select a gut bacterial composition that produces increased SCFAs. These SCFAs influence T lymphocytes in the developing fetal immune system to produce a tolerogenic state that is associated with reduced risk of offspring wheeze and asthma.

Evidence from farm studies, probiotic supplementation, and antibiotic exposure has demonstrated the association between maternal bacterial exposures during pregnancy and offspring asthma. Maternal bacterial exposure in pregnancy influences the developing fetal immune system by a number of potential mechanisms including bacterial exposure of the fetoplacental unit; immunoglobulin-related transplacental transport of gut bacterial components; cytokine signaling producing fetomaternal immune alignment; and transplacental action of gut bacterial metabolites such as SCFAs. Diet is a key determinant of gut bacterial composition and dietary MACs alter fecal bacterial composition to select SCFA-producing bacteria and influence SCFA production. There is consistent evidence for SCFA-mediated effects on the developing fetal immune system; however, an association has not been reported between maternal dietary MACs or SCFA exposure during pregnancy and offspring asthma in humans.

Current measures of MACs, gut bacterial composition, SCFAs, and offspring asthma have limitations. Estimating exposure to MACs and SCFAs is particularly difficult with the proxy measures available. The fecal microbiome, although now nearly ubiquitously used in studies of gut bacteria, must still be interpreted carefully. Developments in lung function testing may allow respiratory phenotype and asthma diagnosis to be more accurately predicted in early childhood, although these techniques still require further validation. Longitudinal birth cohort studies measuring maternal diet, gut bacteria, bacterial metabolite exposures during pregnancy, as well as fetal immune function and offspring asthma to school age are needed to examine the associations identified in this review. Respiratory health is determined by a combination of many antenatal and early-life influences, and it is difficult to predict the relative contribution of any single influence in isolation. Confounding factors influencing respiratory health should be included in any future study order to estimate the magnitude of any effects identified. Asthma is a complex multifactorial condition; however, it appears likely that dietary MACs, gut bacteria, and SCFAs are potential determinants of offspring health. This research holds out the hope of simple, cost-effective interventions in pregnancy to reduce the incidence of offspring asthma.

## Author Contributions

LG is the primary author. The authors have contributed equally to the conception and drafting of this review and are accountable for all aspects.

## Conflict of Interest Statement

This review was conducted in the absence of any commercial or financial relationships, and the authors have no conflict of interest to declare. LG is a PhD candidate at Deakin University for which he receives a scholarship from the University. He has internally submitted portions of this work as part of his Confirmation of Candidature; however, no part of this review has been previously published.
